# Biophysical and structural considerations for protein sequence evolution

**DOI:** 10.1186/1471-2148-11-361

**Published:** 2011-12-16

**Authors:** Johan A Grahnen, Priyanka Nandakumar, Jan Kubelka, David A Liberles

**Affiliations:** 1 Department of Molecular Biology, University of Wyoming, Laramie, WY 82071, USA; 2 Department of Biological Sciences, Carnegie Mellon University, Pittsburgh, PA 15213, USA; 3 Department of Chemistry, University of Wyoming, Laramie, WY 82071, USA

## Abstract

**Background:**

Protein sequence evolution is constrained by the biophysics of folding and function, causing interdependence between interacting sites in the sequence. However, current site-independent models of sequence evolutions do not take this into account. Recent attempts to integrate the influence of structure and biophysics into phylogenetic models via statistical/informational approaches have not resulted in expected improvements in model performance. This suggests that further innovations are needed for progress in this field.

**Results:**

Here we develop a coarse-grained physics-based model of protein folding and binding function, and compare it to a popular informational model. We find that both models violate the assumption of the native sequence being close to a thermodynamic optimum, causing directional selection away from the native state. Sampling and simulation show that the physics-based model is more specific for fold-defining interactions that vary less among residue type. The informational model diffuses further in sequence space with fewer barriers and tends to provide less support for an invariant sites model, although amino acid substitutions are generally conservative. Both approaches produce sequences with natural features like dN/dS < 1 and gamma-distributed rates across sites.

**Conclusions:**

Simple coarse-grained models of protein folding can describe some natural features of evolving proteins but are currently not accurate enough to use in evolutionary inference. This is partly due to improper packing of the hydrophobic core. We suggest possible improvements on the representation of structure, folding energy, and binding function, as regards both native and non-native conformations, and describe a large number of possible applications for such a model.

## Background

Protein-coding sequences play a central role in cellular functions necessary to produce the vast variation in organismal phenotypes observed in nature. To function, most proteins must fold into a unique and stable structure [1]. The structure is responsible for orienting residues necessary for proper function, including binding and catalysis. To maintain fitness, protein function and the underlying structure must be preserved. Protein structure and binding (for example, protein-ligand and protein-protein interactions) are determined by the interactions of individual amino acid residues. Therefore, to mechanistically model the evolution of protein sequences, these residue-residue interactions must be considered, relaxing the common assumption of independent evolution of each amino acid position [2]. Structural models for sequence evolution where function is protein-protein intermolecular interaction will now be considered.

To evaluate if a sequence will fold into a unique and stable structure, it must be demonstrated that the sequence prefers the folded state to both the unfolded state (and folding intermediates) as well as to alternative folded states [3]. From a practical perspective, it is impossible to enumerate the enormous space of all possible alternative conformations [4]. Therefore, sets of representative "decoy conformations" must be used to approximate folding intermediates and/or other stable or meta-stable states [5]. Alternatively, without explicitly considering decoy structures, the inter-residue contacts from different conformations can be randomly sampled [6]. Additionally, a scoring function is needed to quantify differences between the possible states. Strategies developed for generating such scoring functions include those derived statistically from existing structures (informational models, specifically coarse-grained pairwise statistical potentials)[7-9] and those derived from physical first principles [10,11]. Both types of scoring function assume that the native sequence lies close to (within a neutral sequence network of) a thermodynamic optimum, and that there is a gap in energy between the native state and the closest non-native state [12].

Formally, the protein sequence evolution problem relates to the inverse folding problem (whether a sequence will preferentially adopt a particular confirmation) rather than the folding problem. Underlying both problems are similar physical assumptions and similar models [13].

Protein biological function depends not only on proper folding, but also on the ability to bind the target ligands. Therefore, to study sequence evolution, binding must also be evaluated. From a physical perspective this is easier, as the only states to consider are the bound and the unbound states. However, it has recently been suggested that selective pressures on proteins to avoid non-specific binding are also an important aspect of protein fitness, necessitating consideration of binding "decoys" as well [14]. Identifying the actual selective pressures against non-specific binding in a cell is a daunting task, but the number and nature of binding decoys is a major determinant of the ease of evolving new binding interactions. The binding decoy characteristics also affect the level of selective constraint on the binding interface.

Site and function independent models clearly do not capture critical elements of protein evolution. For structure-based models, a good scoring function must produce sequences with similar properties to real proteins. This includes a hydrophobic core that evolves slower than the hydrophilic surface [15]. A dN/dS ratio (the ratio of the nonsynonymous nucleotide substitution rate to the synonymous nucleotide substitution rate) much smaller than unity, particularly for functional residues, should also emerge [16]. More particularly, amino acid substitution rates should show heterogeneity, expressed by a gamma distribution across positions (rejecting an equal rates model of evolution)[17], reflecting their relative importance to folding and function. While the gamma function for rate heterogeneity was adopted for model fit rather than mechanistic purposes, it is one of the most common parameters in standard evolutionary models and accounts for heterogeneity in the substitution rate driven by structural signals as well as other signals in evolutionary sequence data [18,19]. In addition to rate heterogeneity, sequences must also have an energy gap between the native and alternative conformations to ensure rapid and stable folding [20], although structurally disordered proteins represent a distinct class of proteins that do not have this property [21]. The model should place the native sequence near an optimum so as not to provide a signal of directional selection when function is not changing. Consequently, most mutations should be deleterious or nearly neutral rather than adaptive [22]. Lastly, proteins must retain their binding function.

Population size dictates what fitness changes are neutral as well as what mutations become substitutions [23] and must therefore be taken into account. To enable use in forward (simulation) and backward (phylogenetic or population genetic) studies of evolution, especially in a population genetic context, in a complete genome context, or in studies of interactome evolution, the model needs to be coarse grained at a level that allows for sufficient computational speed. In this context the computational cost makes state of the art models and methods from the protein structure community [24] intractable.

Here a novel physics-based scoring function is developed and compared with a commonly used informational approach on the criteria described above. The physics-based model is more specific and predicts less drastic stability changes. Both types of models violate the assumption of high native sequence stability and produce many adaptive mutations through directional selection towards a scoring function optimum, even though the model works with truncation selection. These properties are discussed in light of their effect on modeling accuracy and improvements to the models are suggested.

## Results

Protein folding and function cause interdependence between sites in the protein sequence. For instance, a mutation that removes a cysteine involved in a disulfide bridge (interaction with another cysteine residue at a different position in the protein) is likely to be deleterious, whereas mutation of other cysteines (not involved in disulfide bridge formation) may be more neutral. Of course, disulfide bridges can be lost and interconverted to other types of stabilizing interactions over evolutionary time, albeit with a large transitional barrier due to the strong site interdependence. Such selective pressures can be modeled by scoring the thermodynamic effects of mutations. Two approaches to calculating such scores are the statistically motivated informational methods (specifically, coarse-grained pairwise statistical potentials) and the first principles physics-based methods. Informational models score the likelihood of observing specific types of contacts in the folded protein based on those seen in previously known structures [12]. Physics-based approaches evaluate structures by measuring the fit of residues to geometrical properties such as backbone torsion and residue-residue distances [10]. In both cases the fit to both the native and the many possible non-native conformations must be measured to ensure specificity.

This manuscript sets out to evaluate the ability to replicate biological properties of proteins using both approaches described above through evolutionary simulation. A new physics-based approach is described. Native sequences were evaluated in both approaches to evaluate if they fell within the neutral network of an optimum or if they underwent directional selection initially. Similarly, sequences sampled from within the optimum were evolved to examine the evolutionary properties of each model. With the physics-based model, the relative importance of each term in the folding and the binding functions was evaluated. Together, these analyses give a picture of the evolutionary performance of each model and the appropriateness for use in addressing various questions in molecular evolution.

### The Native State in Physics-based and Informational Scoring Functions

**Figure 1:**
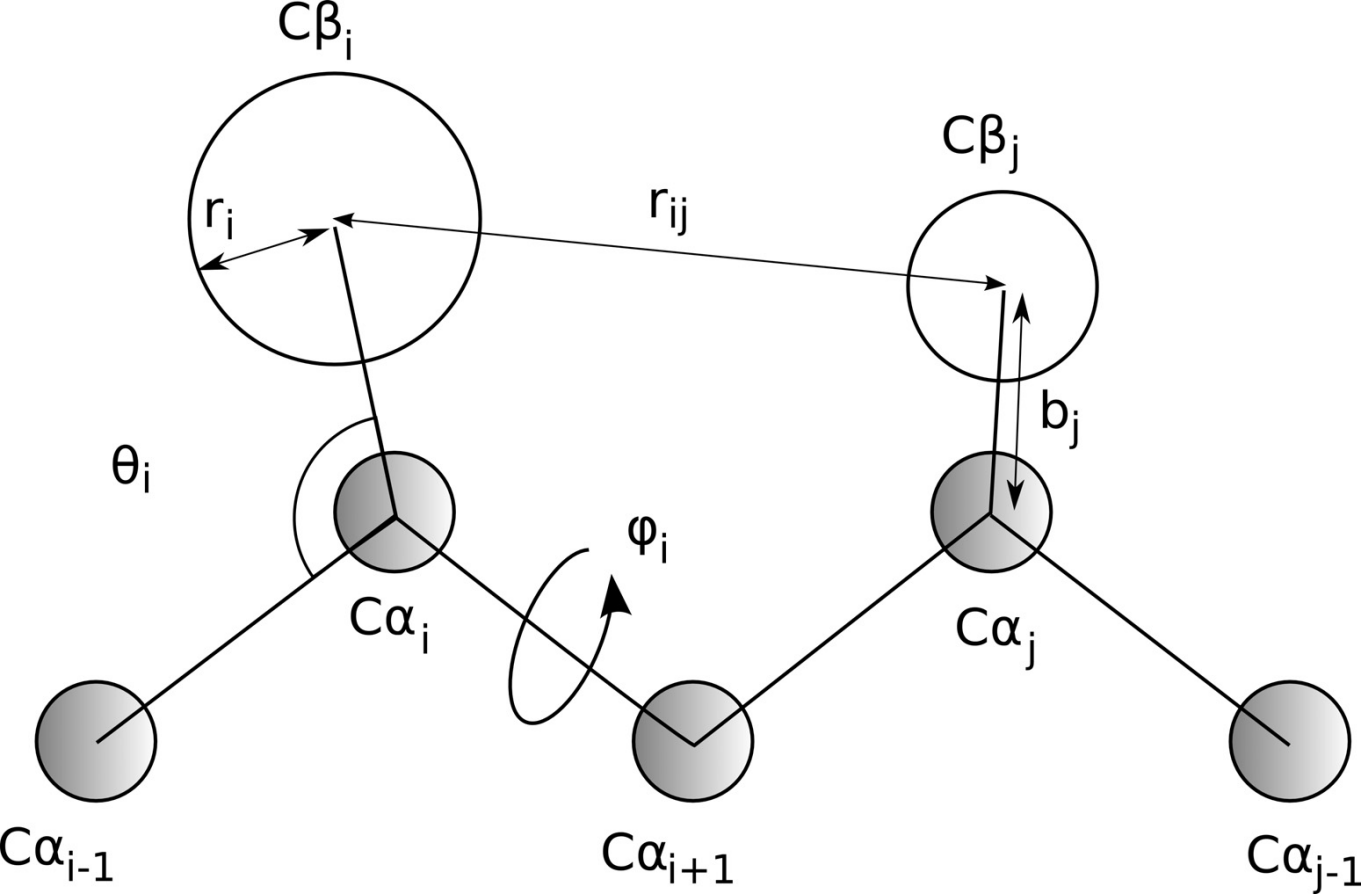
**The two-bead model of protein structure is presented**. Each residue consists of a Cα bead (grey) and a Cβ bead (white). Cα beads are placed at the position of the Cα atoms and form the backbone through being connected by virtual peptide bonds with a torsion angle φ. Each Cα bead (except Gly) binds a Cβ bead with a bond length b and bond angle θ. Cβ beads, whose centers reside at the geometric centroid of the residue atoms, are separated by a distance r_ij _and have a radius r_i_ (proportional to the radius of gyration of the side chain atoms).

In this study a novel physics-based scoring function for a coarse-grained representation of protein structure (Figure 1) was developed. The model (the details of which are described in Methods) includes terms for backbone bond angles, helix and sheet propensities, a new disulfide bridge term, a Lennard-Jones potential to account for van der Waals interactions, a Coloumbic potential, and a solvation potential. The last three terms were also included as part of the binding score function. The newly developed physics-based potential energy function (described in Methods) for a coarse grained protein representation (Figure 1), was evaluated on structure fit to sequence (the fold recognition problem) and sequence fit to structure (the inverse folding problem). A previously developed informational scoring function [9], which is widely used for evolutionary studies was similarly tested. Both models produce an energy gap *Z_fold _*(eq. 14) between native and random conformations for the native sequence for 100 proteins in a structurally diverse test set. Also, both models produce larger energy gaps for the native sequence than random sequences in the native conformation from the same set of proteins: an average native *Z_fold _*of 1.7 for the physics-based model and 4.4 for the informational model. Hence, both model types solve the fold recognition and inverse folding problems to some degree. Binding function can be scored similarly (but on a separate test set of protein complexes, see Methods), and a gap *Z_bind _*(eq. 18) is produced for both models with an average of 0.78 for the informational model and 0.90 for the physics-based model. Not surprisingly, both models score folding (for which they were designed) more specifically than binding.

### Natural protein sequences are not near the modeled thermodynamic minimum

**Figure 2:**
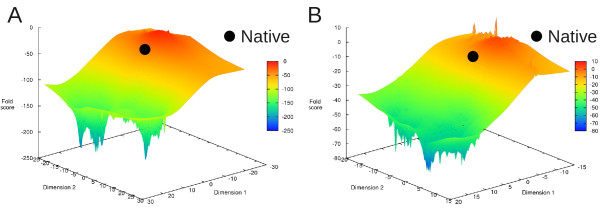
**Folding scores for near-native sequences threaded through the conformation of the SAP protein are shown**. The score distribution is shown as a landscape, with the percentage of the negative native score (i.e *-S_gap_^native^*, see eq. 15) on the Z axis and coordinates in high-dimensional sequence space projected onto the X and Y axes via Sammon mapping. Smaller scores denote more stable sequences (in blue), with larger scores being less stable (becoming progressively more red). The native sequence is marked by a black dot. A) The score distribution for the informational scoring function. B) The score distribution for the physics-based scoring function.

**Figure 3:**
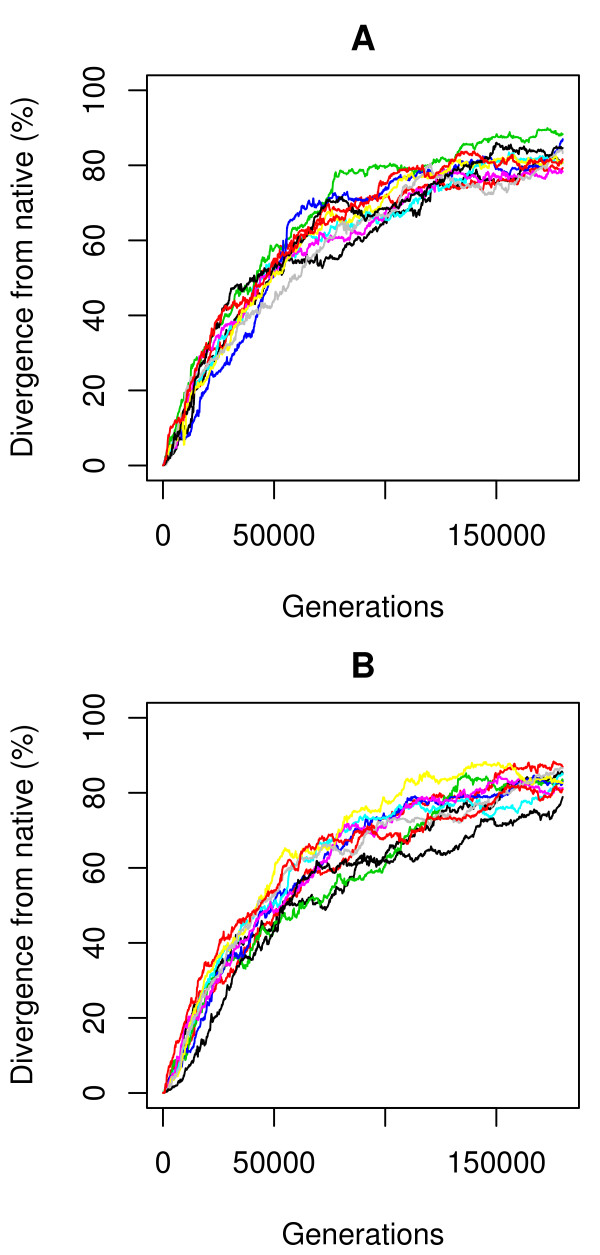
**Sequence divergence of the SAP protein over the simulation time when starting from the native state for the informational (A) and physics-based (B) models**.

However, evaluation of folding stability (in the sense of *S_gap_*, eq. 15) of near-native sequences for a representative protein SAP [25] shows that neither model is highly specific for the native sequence (Figure 2). Within 15% divergence from the native sequence, 42% of sequences are more stable than the native under the physics-based model, and for the informational model this rises to 73%. This is not consistent with biological protein sequences, for which the vast majority of mutations are destabilizing [26]. The sampling procedure also uncovers sequences up to 2.8× more stable than the native within 30% divergence (4.8× for the informational model). The landscapes for both models are qualitatively similar: the native sequence sits on a steep slope and there are local minima elsewhere (Figure 2). The physics-based function yields a somewhat funnel-like landscape, whereas the informational model produces a surface with multiple similar minima. In both cases the overall slope and large fraction of adaptive changes would be expected to cause directional selection away from the native state. Simulations corroborate this by showing rapid divergence from the native state for both models (Figure 3).

### Sequences near the modeled thermodynamic minimum have protein-like properties

**Figure 4:**
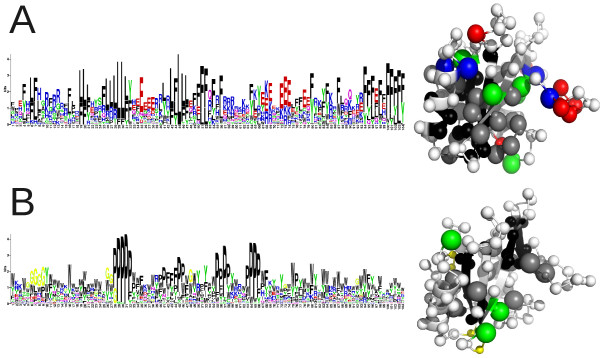
**Highly stable sequences and structures for the SAP protein conformation for the informational (A) and physics-based (B) scoring functions**. Residue frequency distribution in such sequences is shown as a sequence logo (left), and positions with more than 2 bits of Shannon information are mapped onto the structure of SAP with equivalent colors (right, non-informative positions shown in white).

As the native sequence of SAP is not near a stable equilibrium for folding stability on the landscape, stability-biased Markov Chain Monte Carlo (MCMC) sampling (see Methods) was used to find such minima. As seen in Figure 4 (left), these highly stable sequences are very dissimilar to the native state, with only 5% and 11% average identity for the physics-based and informational models, respectively. However, both models show high conservation at many positions and a marked signal for hydrophobicity. The sequences show a rugged energy landscape across sequence space and expectedly complex patterns of change relative to the native sequence (Figure 2).

In the informational model the sequences are dominated by stretches of hydrophobic residues, interspersed with charged residues and a few tyrosines (Figure 4A, left). The remaining positions are highly randomized and do not maintain a specific biochemical character. When mapping the most informative positions onto the structure (Figure 4A, right) a fairly simple pattern emerges. The β-sheet core is dominated by small hydrophobic residues, surrounded by a shell of larger such residues with the occasional tyrosine embedded. Clusters of charged residues maintain the stability of some loops, although most of the highly exposed positions are randomized.

The physics-based model produces qualitatively similar results, but differs in some aspects. Core residues are prolines rather than leucines, and the larger hydrophobic residues surrounding the core tend to be tryptophans instead of phenylalanines (Figure 4B, left). Tyrosines are again favored in semi-exposed positions. However, in place of ionic interactions the physics-based model prefers glycines. Mapping the informative positions onto the structure (Figure 4B, right) reveals that these conserved glycines are located in or near portions of the backbone that are β-sheet-like and highly exposed. Another notable difference is that the physics-based model seems more residue-specific as it conserves fewer positions overall (36 vs 54), but those residues are typically more strongly conserved with larger co-evolutionary barriers to change. Both models impart roughly the same selective pressure overall.

### Evolutionary simulations near minima reproduce biological sequence properties

**Figure 5:**
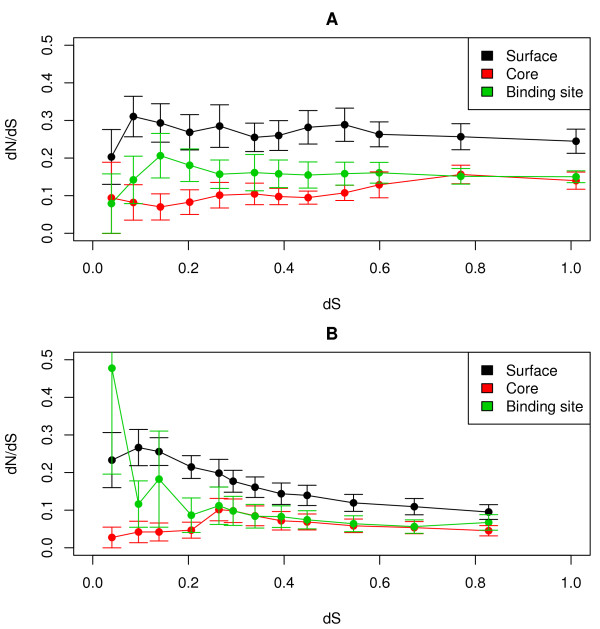
**dN/dS vs dS (proportional to simulation time) for surface (black), core (red) and binding site (green) residues is shown**. Values reflect the mean of the most common allele in each population, with standard error bars indicating inter-population variation. Simulations using the informational scoring function in A), simulations using the physics-based scoring function in B).

**Figure 6:**
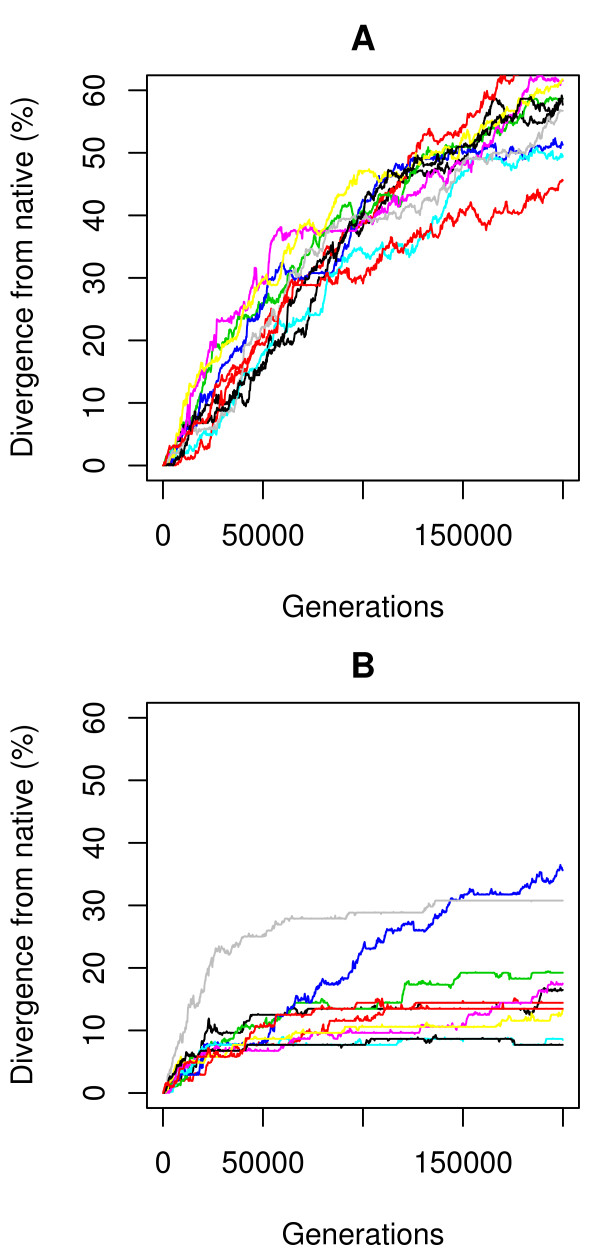
**Sequence divergence over the simulation time when starting from a stable state the SAP protein structure is shown for the A) Informational model and the B) Physics-based model**.

Using the stable sequences as starting points for population-based simulations allows comparison of the models with respect to other properties than folding stability. As can be seen in Figure 5, evolutionary rate ratios in both cases are comparable to those found in real orthologous proteins (dN/dS of 0.1-0.5) and indicative of the negative selective pressure applied (dN/dS < 1)[16]. The rates also vary as expected depending on the position of each residue in the 3D structure. Exposed surface positions evolve up to three times faster than the buried and highly constrained core residues. Residues in the binding site, which are exposed but under functional selective pressure, show an intermediate rate. Interestingly, under both models the rates in the selectively important regions (core and binding site) become very similar over time. The physics-based model consistently shows lower rates throughout the structure, with the exception of initial changes in the binding site. Surface mutations are noticeably more selected against compared to the information-based approach, which is consistent with the explicit consideration of solvation in this model. The physics-based model also restricts movement on the sequence-stability landscape to one or a few well-defined (but very large in sequence space) basins, whereas the informational model appears to diffuse more neutrally across a mostly flat surface (Figure 6).

Real protein sequences also show rate heterogeneity across positions (which is modeled by a gamma distribution of rates of amino acid substitution per site in statistical comparison with equal rates across sites), such that most positions evolve slowly but some evolve quite rapidly [17]. The fit of such a model to the simulated sequences was found to be reasonable (Table 1). The relative support for including gamma-distributed rates is generally high using the informational model, and somewhat lower in the physics-based model. The latter instead shows higher relative support for a model with some invariant sites among common parameters in site-independent substitution models. The drop in support for rate heterogeneity toward the end of the simulations may indicate emergence of rate heterotachy [27], caused by migration to a non-native stable basin through intramolecular co-evolution. This is consistent with a view of proteins where an initial equal rates across sites model with a small number of substitutions gives rise to a gamma distribution as substiutions accumulate, driven by the three dimensional structure. With further substitution, intramolecular co-evolution gives rise to support for a covarion process rather than for a gamma distribution [28]. When gamma is supported, the shape parameter of the gamma distribution (0.1-3.0) is consistent with those found in biological sequences in general [29] and with the shape parameter 1.83 obtained from the Pfam SH2 domain seed alignment in particular.

**Table 1:** The protein-like properties of sequence evolution over simulation time is shown

Simulation time	Model	Core	Surface	Gamma Support	Shape Parameter
		Hydrophobicity	Hydrophobicity		
		(%)	(%)		
**5000**	Informational	39.7	11.2	0.35	0.02

**25000**	Informational	42.1	15.2	0.72	0.95

**50000**	Informational	42.9	18.5	0.72	1.46

**70000**	Informational	45.0	20.4	0.69	1.55

**100000**	Informational	47.4	23.0	0.73	2.07

**150000**	Informational	47.7	27.7	0.49	2.61

**200000**	Informational	49.6	30.5	0.12	2.73

**5000**	Physics-based	83.4	49.2	0.20	3.31

**25000**	Physics-based	82.8	49.5	0.37	2.58

**50000**	Physics-based	81.7	49.1	0.31	2.06

**70000**	Physics-based	81.7	48.4	0.61	1.86

**100000**	Physics-based	81.1	48.6	0.09	1.87

**150000**	Physics-based	81.6	48.2	0.25	1.76

**200000**	Physics-based	81.1	48.0	0.10	1.88

Simulation time is listed in generations. Core and surface hydrophobicities shown reflect the population average percentage of hydrophobic residues in buried and exposed positions. Gamma support indicates the fractional support for a model including a gamma distribution of rates, and the shape parameter shows the α value of such a distribution.

**Figure 7:**
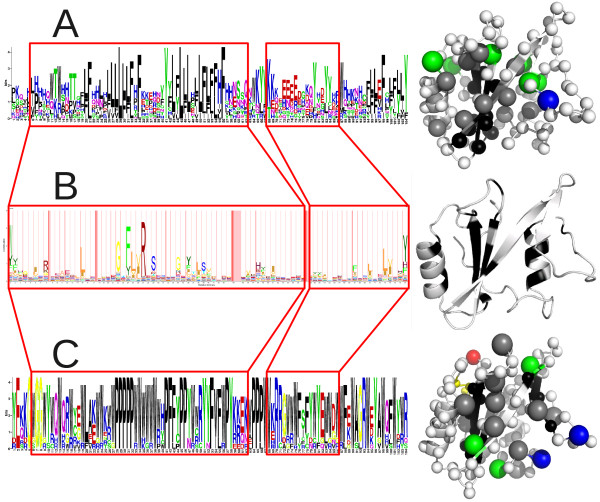
**A comparison of simulated sequences and structures with those of known SH2 proteins is shown**. A) and C) depict sequences simulated under the informational and physics-based models, respectively, while B) depicts the SH2 family from the Pfam database. Residue frequency distributions are shown on the left as sequence logos for A) and C), and as HMM emission probabilities for B). Red boxes indicate matching positions between the sequences. For A) and C), positions with more than 2 and 3 bits of information, respectively, were mapped onto the SH2 structure on the right with equivalent colors (white beads indicate less informative positions). For B), the 25 most probable positions were colored black on the backbone (remaining positions again in white).

The sequences and structures produced by each simulation (Figure 7) further highlight the differences between the models. The sequence collection from the informational model with truncation selection on folding and binding (Figure 7A) shows the same basic features as those found by sampling from stable structures (Figure 4A, left) but with added emphasis on tyrosines rather than ionic interactions. There is some indication of increased conservation of the binding site (around positions 52, 68, and 92). During the simulations core hydrophobicity increased by ~25%, and surface hydrophobicity almost tripled (Table 1). This resulted in a similar ratio of core and surface hydrophobicities as in the native SAP sequence (1.63 and 1.80, respectively). By mapping conserved positions onto the structure (Figure 7A, right) we recover the starting pattern (Figure 4A, right) of conserved β-sheet core, a shell of larger hydrophobic residues around this, and tyrosines at medium exposure.

As noted above, the physics-based model sampled with truncation selection for folding and binding is substantially more restrictive in how many non-synonymous substitutions it allows, and the simulated sequences reflect this (Figure 7C, left). Nearly 1/3 of the positions are effectively invariant over shorter evolutionary timeframes, compared to only a few residues in the informational model. Structural mapping of highly conserved positions (Figure 7C, right) again recovers the starting pattern (Figure 4B, right), although a few β-sheet-associated glycines have been replaced by charges on the most exposed loops. The binding site is very highly conserved. Only very exposed positions (17-18, 59-62, etc.) have started to randomize, mostly containing polar and charged residues. Core and surface hydrophobicities started close to those of the native sequence (83% and 50% vs 72% and 40%), and did not change appreciably during the simulation (Table 1).

Comparing the simulated sequences to the Hidden Markov Model of the Pfam [30] SH2 family (Figure 7B), some similarities are noticeable. Both models reproduce the overall conservation of the core residues, particularly the very buried ones. The informational model is more accurate in predicting the residue type within the core (leucines and phenylalanines), whereas the physics-based model correctly conserves some glycines. Neither model captures the defining functional feature of the family (a conserved arginine in the binding pocket), but this is due to the SAP SH2 domain binding a non-phosphorylated ligand. Instead the models more generally preserve residues within the binding interface. This type of conservation is absent in the HMM since SH2 domains have unique specificity-determining binding site geometries. Similarly, since the HMM reflects several dozen distinct structures and sequences it is less conserved overall than simulated sequences derived from a single structure evolved over relatively short evolutionary distances.

### Components of the physics-based model have variable importance for scoring

**Figure 8:**
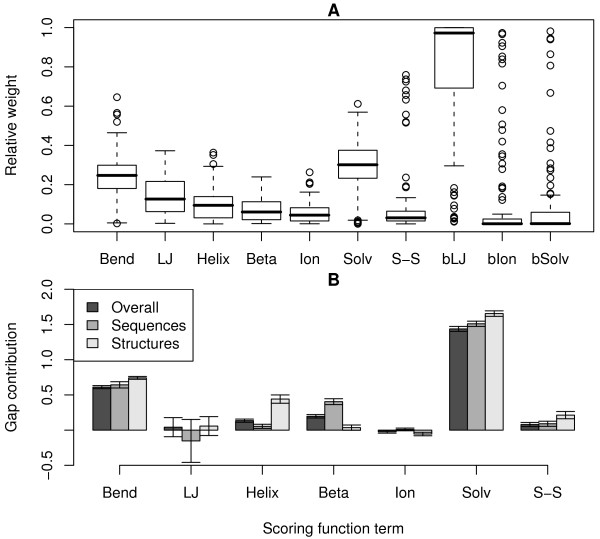
**The importance of terms in the physics-based scoring function is shown**. A) The distributions of relative weights for each term in the full scoring function on proteins/complexes in the test sets (binding terms prefixed with "b") are depicted. Outliers are shown as empty circles. B) The gap between the native score and the distribution of random samples when using only single terms in the scoring function is depicted. Gaps are shown for the set of random sequences (dark grey), the set of random geometries (light grey) and the combined set (black). Note that only folding score terms are shown.

The parameters of both models would be expected to have an influence on the sequences produced. The properties of the informational model have been described elsewhere [9]. Here, the nature of the physics-based model is examined in more detail. The term weights are re-adjusted for each individual protein in the test set, and the resulting distributions are shown in Figure 8A. Solvation, side chain angles (bend) and pair-wise interactions (LJ) are quite important, consistent with a well-packed hydrophobic core with biological side chain rotamers and no steric clashes. Buried salt bridges (ion) and disulfide bonds (S-S) seem less influential, but are also comparatively rare in the investigated protein structures. Of the secondary structure terms, helix propensity is somewhat more specific than β-sheet tendency. For binding, shape complementarity (bLJ) is by far the most effective term. However, these parameter values are found in the context of the complete scoring function and may have strong synergistic effects in combination with other terms. An examination of the contribution of each term in isolation to the total gap between native and random states (Figure 8B) reveals some additional trends. The LJ term is nearly uninformative on its own, indicating that it needs to be combined with at least one other term to be specific. The difference between representations of the non-native state is visible in the helix and beta terms. The helix term is more informative when considering the gap to decoy structures, whereas the beta term is more effective in discriminating against random sequences.

**Figure 9:**
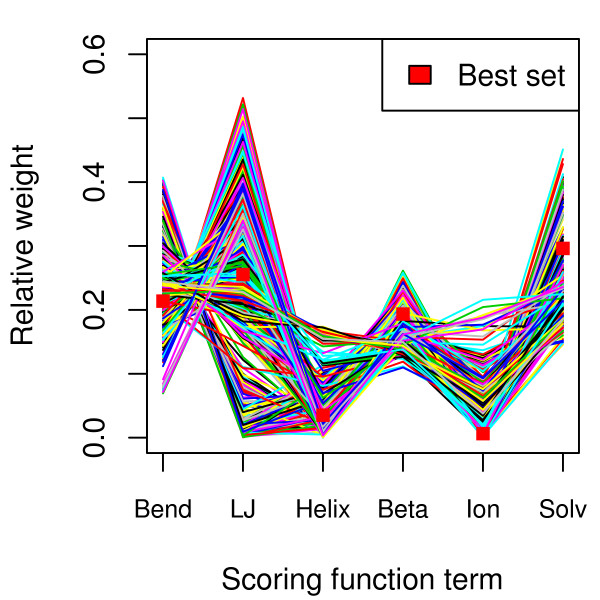
**Parameter sets of approximately equally likely term weights for the SAP protein are shown**. Each set is represented as a colored line, and the best set is shown as filled red squares. The Cys-Cys term was not included because of the lack of disulfide bridges in the SAP protein, although there is potentially limited information in that term in contrasting with alternative conformations with disulfide bridges.

Repeating the parameterization step many times on the SAP protein reveals further differences between the terms. Although the algorithm converges on roughly the same optimality score (it is numerically stable), there is substantial variation in parameter values. The co-dependence of roughly equally likely solution sets for term weights is shown in Figure 9. Some terms (bend and solvation) have pronounced optima around which there is little variation, but others (ionic, LJ, helix) produce a relatively flat optimality landscape. Particularly the less important terms have weights that vary over several orders of magnitude. This can cause difficulty with finding realistic stable sequences for a particular fold, as small changes in some terms (notably the ionic term) can have large effects on the sequence composition. A representative sample of parameter sets was used to generate collections of stable sequences, and the most protein-like set was selected (Figure 9, filled squares). The parameter values fall close to the center of each distribution, although charges are de-emphasized and solvation has increased importance.

## Discussion

In evaluating our ability to model the evolution of sequences using structural and functional (binding) constraints, both informational and physical models show protein-like properties, but also need further improvement. Despite a stability gap between random sequences (and structures) from the native for both methods, the native sequence is not near enough to a thermodynamic stability optimum to prevent directional selection. Sequences near such an optimum are more homopolymeric in composition. The evolution of such sequences, however, does show many attributes of real protein evolution.

The effect of negative (purifying) selection, a dN/dS ratio smaller than 1, is well modeled by both approaches, even reproducing the known correlation between exposure and evolutionary rate [31]. The physics-based model produces the stronger selective pressure overall, particularly so on the protein surface (due to the solvation term). Variation in this rate across positions is mimicked by the informational model, which more consistently supports a gamma parameter throughout the simulation. The physics-based model instead conserves some positions completely over shorter evolutionary distances, but ultimately supports a covarion process rather than simply rate heterogeneity. In particular, hydrophobic residue content is much closer to that of the native sequence. When it comes to the exact identity of such hydrophobic residues (e.g. leucines vs prolines) the informational model has higher correspondence to native residues, presumably due to being derived from known sequence/structure combinations. Overall the physics-based model appears more specific for local but crucial contributions to free energy of folding, whereas the informational model produces sequences with protein-like properties but without fold specificity.

In the context of fold specificity, a major difference between models is consideration of secondary structure. The physics-based model is enriched in prolines in the β-sheet core, and glycines in other sheet-like portions of the backbone. In principle any small hydrophobic residue should suffice in the tightly packed and highly buried core positions, but prolines in particular score well in the helical term (being helix breakers in the most non-helical segment of the protein)[32] and are mostly acceptable in the sheet term. The glycines in half-exposed positions with sheet-like geometry provide an excellent compromise between the helix, sheet and solvation terms, and have little effect on the remainder of the scoring function. Although somewhat unrealistic in the context of the native sequence, this does demonstrate the strong impact of the particular backbone conformation under selection.

The physics-based model is also different in that its parameters are adjusted for each protein. This is beneficial in adding specificity to the model [33], but also presents issues with parameter selection [34]. The Lennard-Jones term in particular is surprisingly variable and offers little benefit in isolation. When testing against random sequences, this is mostly due to the side chain replacement protocol. It minimizes a Lennard-Jones function [35], thereby removing much of the signal a priori. Side chain optimization with a complete scoring function including other terms might remedy this problem. The vastly larger utility in a binding context (Figure 8A), where no such pre-minimization occurs, further illustrates this point. The test against random compact structures with constant sequence (Figure 8B) is more illuminating. It shows that the L-J term needs the restrictions imposed by other factors (for example, solvation and secondary structure) to efficiently identify a protein-like heteropolymer. In other words, it adds information to the total function, but only when the solution space is already somewhat restricted.

Generally, the parameterization of both folding and binding scoring functions is fairly difficult. For instance, the choice of the reference state (the unfolded or unbound molecules) has a substantial impact on the calculations. The ruggedness of the optimality landscape in parameter space makes it quite difficult to find the global optimum, and parameterizing folding and binding functions separately may lead to inconsistencies between the two. Future work will explore more sophisticated approaches to solving these problems.

The specificity of either model is ultimately reflected by the shape of its stability landscape. As seen both near the native sequence (Figure 2) and non-native optima (Figure 6), the physics-based model produces a landscape that is more rugged and has fewer equivalent minima. This is consistent with theoretical expectations of the real free energy landscape in both conformational [3] and sequence [36] space. The result is that the simulated sequences tend to be more conserved, stay near the starting ("native") point during simulation, and show mostly deleterious or neutral mutations. In contrast, under the informational model populations simply diffuse across a relatively flat sequence landscape with less co-evolutionary pressure between interacting sites, rapidly moving away from the starting point. In this aspect the physics-based model shows more protein-like and fold-specific behavior overall, despite selecting sequences with some compositional "quirks", as discussed above. Informational methods, especially those built only on pairwise contacts, necessarily lack specificity. For example, all Lys-Phe interactions are not equivalent. Those oriented in a plane will be repulsive, while those that are orthogonal will generate a cation-pi effect and be attractive. This is but one example. Physical models, at the right level of granularity, will not suffer from this problem and are thereby inherently attractive for this interaction specificity. Further, they enable study of the forces driving evolutionary processes at a physical level.

Why then are the native states so far from sequence optima? The answer is found in the approximations made by the models. Packing is perhaps the most severely affected, as can be seen from the accumulation of large hydrophobic residues in both models. These substitutions fill up the empty space that results from the coarse-graining procedure. Spherical Cβ beads, for instance, are a particularly poor approximation for elongated (Lys, Arg, etc.) or flattened (Trp, Phe, etc.) residues. A structural model with more than one bead per side chain, such as that of Hills and co-workers [37], could remedy this issue. The representation of ions as spherical point charges is also problematic, and salt bridge formation could be better addressed by using a model of induced dipoles [38]. Many entropic contributions are not considered, although the physics-based model includes some of them implicitly in the solvation term. In addition to entropic considerations, explicit hydrogen bonding [39], cation-pi interactions [40], and other interactions are not considered, but would increase model complexity. Finally, an overarching problem may be found in the effect of individual mutations. A typical disadvantageous mutation under the informational model has a very small effect on the stability score *E(s, c) *(eq. 12) (< 0.5 kT units out of tens of kT units, where k is Boltzmann's constant and T is the absolute temperature), and the physics-based model generally predicts even smaller stability changes. In real proteins the average mutation is strongly deleterious (3 kcal/mol out of a total ΔG of ~10 kcal/mol, where ΔG is the free energy difference between the unfolded and folded protein structure)[41], indicating that the models are much too lenient when it comes to the cost of mutation. Appropriate weighting of contributing terms or more precise modeling of packing would improve this aspect, and make the energy landscape more realistically rugged.

Early attempts at considering alternative states in the above models of folding used explicit decoys. Because of the large number of potential alternative states for a sequence, both kinetic traps and thermodynamic optima, evaluating even a large finite number of such states proved inadequate. Further, identifying the states that were most informative near the native confirmation was not clearly driven by fold sequence or geometry and it may be that the relevant structures originate from smaller regions of diverse structures. The random contacts model [6] proved powerful and efficient in sampling many contacts from diverse structures as an implicit solution.

For binding, there is a greater conundrum in that while the real biological decoys are not known, a random contacts model has the potential to be too restrictive in selecting against binding interactions that are not biologically deleterious. For an alternative binding interaction to be deleterious, the proteins need to have a deleterious interaction (as defined by biological fitness) and to have the potential to interact at the same time, in the same cellular location, at the right concentration [42]. However, the proper decoys are frequently not known and this restricts the use of the alternative approach of explicit binding decoys.

Further, when evaluating binding specificity as a function of affinity it is assumed that complex thermodynamic stability (and relatedly, life time) must be maximized. While this is a relevant constraint for cellular structures such as the nuclear pore complex [43], protein complexes involved in signaling or even in forming the cytoskeleton have transient associations that are necessary for functional signaling [44-46]. A requirement of transient binding means that dissociation kinetics are selected to be just fast enough, not as slow (corresponding to very stable complexes) as possible. Therefore, even in the limit of perfect predictions one should not expect to recover the native binding site sequence based on thermodynamics alone.

From the perspective of protein structural space [47], the folding decoys all lie near the native conformation. The total amount of secondary structure is conserved, as are the relative percentages of helices and sheets. This means that the decoys can be thought of as being sampled from the same Superfamily, or at least Fold, in the SCOP hierarchy [48]. Conservation of residue exposures and sequence length also guarantees that the conformations have roughly the same radius of gyration as the native state. This should cause selection against subtle misfolds and accumulating pathway intermediates, but may be a poor representation of the diversity of conformations a sequence may fold into and thus may miss important local alternative conformations that are close in energy. For example, an increase in residues with high helical propensity could shift the lowest-energy conformation toward an all-α topology instead of α+β. The current approach does not allow modeling this type of event, but evaluation of the partition function of the Boltzmann probability of folding is a notoriously difficult (and currently unsolved) problem [49]. A critical aspect of characterizing the energy landscape is the role of decoy structures in restricting the funnel shape as well as providing fold specificity, and this is a challenging task.

Recently, the utility of site-interdependent structural models of sequence evolution was evaluated in the context of phylogenetics [50-52]. Despite use of a variety of scoring functions and probability measures, the utility of such models was found to be generally low. Minimizing a coarse-grained potential failed to recover much of the native sequence and did not outperform site-independent models [51]. Simulating the effects of substitutions on folding energy revealed a rapid divergence from the native state even when modeling solvation in addition to residue-residue contacts [50], and including the contribution of folding energy to transition path probability can even decrease tree reconstruction accuracy [52]. These results are all consistent with the nature of the coarse-grained energy landscape seen above. There is no global minimum near the native sequence, and its position on a steep slope results in rapid divergence in terms of sequence, energy or both. Models that accurately characterize the structural effects of substitutions are urgently needed to make progress in this direction. It is also worth noting that these approaches all relied on informational potentials, which we have shown above to be less structurally specific than physics-based scoring.

With models that properly characterize the evolution of proteins, many important evolutionary biological questions can be addressed. What are the patterns of sequence evolution associated with different mechanisms of duplicate gene retention? Relatedly, how easily does orthologous neofunctionalization occur and how dependent upon protein fold and binding interface size is it? What are the roles of positive and negative pleiotropy in restricting neofunctionalization? How do structural transitions occur between neighboring folds? At the contact level, how are different stabilizing interactions interconverted (for example, the transition between Cys-Cys, cation-pi, and Coulombic interactions)? What is the interplay between population size, fold distribution, and functional evolution? To address these important evolutionary biology questions, continued progress on models such as described here is needed.

## Conclusions

Proteins evolve according to the laws of physical chemistry, biochemistry, and population genetics. Classic phenomenological site independent models, while computationally simple to use, do not adequately describe evolutionary processes for either phylogenetic or functional evolutionary analysis (comparative genomic) purposes. Mechanistic models built upon the type described here will be necessary. Biological (for example, regulatory or metabolic) pathways dictate the level of constraint on physical constants and such considerations will also need to be taken. While the currently available models remain inadequate, an understanding of the problems in physical chemistry associated with evolutionary models will lead to future improvements, with many downstream applications.

## Methods

### The two-bead model

The protein structure is represented by a reduced two-bead model originally described by Levitt [53] and more recently used by Mukherjee and Bagchi [54] as well as Grahnen et al. [35]. Each residue is represented by one backbone bead (Cα_i_) and one side chain bead (Cβ_i_) (Figure 1). The Cα bead is centered on the Cα atom in an all-atom representation and has a radius of 1.8 Å. For a residue *i *(except glycine), the Cβ_i _bead center is placed at a distance *b_i _*from Cα_i_, which is determined as the centroid of all the side chain atoms (including the Cα_i _atom), and assigning a residue-dependent radius *r_i_*. Glycine is simply represented with a Cα bead with the appropriate properties (hydrophobicity etc.). For residue *i*, where 2 < = *i *< = *N*-2 in a protein with *N *residues, we define *θ_i _*as the angle between Cα_i-1_, Cα_i _and Cβ_i_, and *φ_i _*as the torsion angle between Cα_i-1_, Cα_i_, Cα_i+1 _and Cα_i+2_. Residues within two bonds of the termini do not have a defined *φ_i_*, and the residues at the N-terminus do not have a defined *θ_i_*. Finally, *r_ij _*describes the distance between the center of any two beads *i *and *j *in the model. In the informational representation of protein structure *r_ij _*was measured by taking the minimum distance between the Cβ beads in residues *i *and *j *(Cα is substituted for Cβ for glycines).

### Threading sequences into conformations

To thread a sequence *s *into a protein backbone conformation *c*, the side chain replacement algorithm SARA [35] was used. SARA is a very fast approach based on iterative Markov Chain Monte Carlo refinement of the replaced side chain geometry. Backbone coordinates were not adjusted during the replacement procedure, and *c *was assumed to be constant throughout.

### Scoring protein folding with the physics-based model

To evaluate the fit of a sequence *s *to a backbone conformation *c*, where the number of residues *N *is the same for both *s *and *c*, *s *is threaded into *c *(see above) and then a weighted scoring function *V(s, c) *is computed:

(1) $$V\left(s,\;c\right)=w_{bend}V_{bend}+w_{LJ}V_{LJ}+w_{helix}V_{helix}+w_{beta}V_{beta}+w_{ion}V_{ion}+w_{solv}V_{solv}+w_{S-S}V_{S-S}$$

where:

*V_bend _*is a harmonic bending potential around the equilibrium bond angles for Cα-Cβ bonds:

(2) $$V_{bend}=\left(1∕2\right)K_{\Theta }\overset{N}{\underset{i=2}{\sum }}{\left(\Theta_{i}-\Theta_{0}^{t}\left(i\right)\right)}^{2}+\left(1∕2\right)K_{\Theta }\overset{N-1}{\underset{i=1}{\sum }}{\left(\Theta_{i+1}-\Theta_{0}^{t}\left(i\right)\right)}^{2}$$

*K_Θ _*is the force constant for the bending potential (10.0 kJ mol^-1 ^rad^-2^), *Θ_i _*and *Θ_i+1 _*are defined as described above, and $\Theta_{O}^{t}$ is the equilibrium bond angle for a Cβ bead of type *t*.

*V_LJ _*is the pair-wise vdW interaction potential, approximated as a sum of Lennard-Jones potentials:

(3) $$V_{LJ}=4\sum_{i,j}\varepsilon_{i,j}[{\left(\frac{\sigma_{ij}}{r_{ij}}\right)}^{12}-{\left(\frac{\sigma_{ij}}{r_{ij}}\right)}^{6}]$$

*ε_ij _*is the interaction parameter between beads *i *and *j *(a pair of Cα beads, a pair of one Cα bead and one Cβ bead or a pair of two Cβ beads), based on their respective hydropathy indexes, *σ_ij _*is the collision diameter of beads *i *and *j *(i.e. the sum of their radii), and *r_ij _*is the defined in Figure 1. The sum *ij *runs over all pairs of beads noted above.

*V_helix _*is the helical potential, an approximation of the entropic and steric effects of the backbone forming an α-helix:

(4) $$V_{helix}=\sum_{i=3}^{N-3}\left[\frac{1}{2}K_{i}^{1-3}{\left(r_{i,i+2}-r_{h}\right)}^{2}+\frac{1}{2}K_{i}^{1-4}{\left(r_{i,i+3}-r_{h}\right)}^{2}\right]$$

*K_i_^1-3 ^*is average helical propensity of residues *i*, *i+1 *and *i+2*. *r_i, i+2 _*is the distance between Cα beads *i *and *i+2*. *r_i, i+3 _*is the distance between Cα beads *i *and *i+3*. $K_{i}^{\text{1 - 4}}$ is the average helical propensity of residues *i*, *i+1*, *i+2*, and *i+3*. Finally, *r_h _*is the equilibrium helix inter-bead distance (5.5 Å).

*V_beta _*is the beta-sheet potential, constructed analogously to *V_helix _*but using an equilibrium beta torsion angle instead of a bead-bead distance:

(5) $$V_{beta}=K_{i}^{1-4}\overset{N-2}{\underset{i=2}{\sum }}{\left(C_{b}\left(\varphi_{i}-\varphi_{b}\right)\right)}^{2}$$

$K_{i}^{\text{1 - 4}}$ is the average beta propensity of residues *i-1*, *i*, *i+1*, and *i+2*. The beta propensity for a given residue type is constructed using the same linear scaling of helical propensities used by Mukherjee, but is based on Kim and Berg's beta propensity scale [55] rather than Pace and Scholtz's helix propensity scale [32]. *C_b _*is a scaling constant (0.01) selected to scale the range of torsion angles to a similar magnitude as the range of bead-bead distances in *V_helix_*. Torsional angle *φ_i _*is defined above (Figure 1), and *φ_b _*is the equilibrium beta sheet value (210°) for a two-bead representation as described by Bahar and coworkers [56].

*V_ion _*is an electrostatic potential based on Coulomb's Law:

(6) $$V_{ion}={\text{C}}_{\text{c}}\underset{i,j}{\sum }\frac{q_{i}q_{j}}{\varepsilon r_{ij}}$$

*C_c _*is a scaling constant (1,000) selected to scale the term to similar magnitude as other terms, *q_i _*and *q_j _*are the charges of residues *i *and *j *(+1 or -1 depending on residue type), *r_ij _*is the distance between beads *i *and *j*, and *ε *is the dielectric constant of the protein interior (3.0)[57], with the sum running over all pairs of charged residues with a SASA (see below) of less than 0.25. This roughly approximates the strong screening of charged interactions due to the highly polarizable surrounding water, and the nearly negligible effects of the largely non-polarizable interior of the protein [58]. The whole term is then scaled by a weighting term *w_ion _*when used in *V(s, c) *(eq. 1), so *C_c _*has no effect on the parameterization, but is necessary computationally so as not to introduce errors due to lack of floating point precision.

*V_solv _*is the potential due to solvation of the protein, approximated by a novel implicit solvent model based on solvent-accessible surface area (SASA):

(7) $$V_{solv}=\overset{N}{\underset{i=1}{\sum }}h_{i}SASA\left(i\right)+p_{i}\left(1-SASA\left(i\right)\right)$$

*h_i _*is the hydrophobicity-based interaction parameter of residue *i *(equivalent to *ε_ii _*above) and *p_i _*is the "polarity index", constructed as *p_i _*= (*h_max_-h_min_*) - *h_i_*. *SASA(i) *is the fraction of solvent-accessible surface area of residue *i *calculated by the NeighborVector method of Durham [59] as adapted for the two-bead representation.

*V_S-S _*represents the stability contribution due to formation of predicted disulfide bonds:

(8) $$V_{s-s}=\underset{i,j}{\sum }f\left(r_{i,j}\right)$$

where

(9) $$f\left(r_{i,j}\right)=\{\begin{array}{cc} -1, & r_{i,j}<r_{ss}\\ 0, & otherwise \end{array}$$

and *r_ij _*is the distance between two cysteine Cβ beads and *r_SS _*is the maximal Cβ-Cβ distance in a typical disulfide-bonded cysteine pair (4.5 Å[60]). The sum *ij *runs over all pairs of cysteine residues. This form of *V_cys _*predicts disulfide bonds with a specificity of 0.98, sensitivity of 0.91 and Matthews Correlation Coefficient of 0.79 as measured on the structural data set used to construct SCWRL 3.0 [61].

Finally, *w_x _*are weights for each individual term in eq. 1, determined in a procedure described below.

### Scoring protein-protein interactions with the physics-based model

When using protein-protein interaction as a measure of protein function, it becomes necessary to evaluate the strength of the interaction, a proxy for the free energy change of binding or the dissociation constant. The non-covalent terms from the folding scoring function were adapted to compose an interaction score *V(s_1_, c_1_, s_2_, c_2_) *between a sequence *s_1 _*in conformation *c_1 _*and a sequence *s_2 _*in conformation *c_2_*:

(10) $$V\left(s_{1,}c_{1,}s_{2,}c_{2}\right)=w_{LJ^{\prime }}V_{LJ}^{\prime }+w_{ion},V_{ion}^{\prime }-w_{\Delta solv}V_{\Delta solv}$$

$V_{LJ}^{\prime }$ and $V_{ion}^{\prime }$ are the same as above but evaluated for inter- rather than intra-molecular bead pairs.

*V_Δsolv _*is the change in solvation potential upon binding:

(11) $$V_{\Delta solv}=V_{solv}\left(s_{1,}c_{1}\right)+V_{solv}\left(s_{2,}c_{2}\right)-V_{solv}\left(complex\right)$$

where *complex *is the bound state. The weights *w_x _*are determined as described below.

### Scoring protein folding and interactions with the knowledge-based model

The approach of Bastolla and co-workers [9] to was used to score folding under a knowledge-based model, and adapted to scoring protein-protein interactions. Briefly, a sequence *s *in conformation *c *has a folding score of

(12) $$E\left(s,\;c\right)=\sum_{i,j}U_{ab}\left(i,j\right)C\left(i,j\right)$$

where *U_ab _*is a matrix describing the free energy gain when amino acids of type *a *and *b *are in contact, *C *is the contact map of *c *(1 when the Cβ beads of i and j are closer than 4.5 Å, 0 otherwise) and the sum runs over all residue pairs (*i, j*) that are separated by more than four residues in primary sequence.

To evaluate a protein-protein interaction, a protein complex score is calculated as

(13) $$E\left(s_{1},\;c_{1},\;s_{2},\;c_{2}\right)=\sum_{i,j}U_{ab}\left(i,j\right)\;C\left(i,j\right)$$

where *C *is evaluated between *c*_1 _and *c*_2 _(inter-molecularly), and the sum runs over all residue pairs (*i*, *j*) such that *i *comes from *c_1_* and *j *comes from *c_2_*.

### Calculating folding and binding specificity

Folding and binding specificity were evaluated with several considerations. For example, with folding, a given sequence should have the specified backbone rather than an alternative as its most stable state and also present a gap between this state and unfolded or misfolded states. The size of this gap can be measured as a Z-score [62-64]:

(14) $$Z_{fold}=\frac{\left\langle G\right\rangle -G_{nat}}{\sigma \left(G\right)}$$

where ***G ***is the distribution of free energies (ΔG) in the alternative conformations, *G_nat _*is the free energy of folding into the native conformation, and <***G***> and *σ(**G**) *describe the location and dispersal of that distribution. In other words, *Z_fold _*can be interpreted as measuring the specificity of a protein sequence for its native structure, or equivalently a combination of unfolding and misfolding stability as well as the preference for the specified fold over alternative folds.

During simulation and sequence sampling, *G_nat _*is approximated by *V(s, c) *(eq. 1) or *E(s, c) *(eq. 12) for the current sequence and a native conformation, and **G **by calculation of the same measure for 100 random decoy conformations. The decoy conformations were generated by independently randomizing each type of geometrical measurement in the native structure, in accordance with the Random Energy Model (REM) of Bryngelson and Wolynes [6]. For the informational model this means randomizing the residue-residue contacts, whereas in the physics-based model the angles, distances, etc. involved in each term are shuffled. The fold score gap is then calculated as

(15) $$S_{gap}=\left\langle G\right\rangle -G_{nat}=\sigma \left(G\right).Z_{fold}$$

where <***G***> is estimated by the median of ***G***. The dispersal *σ(**G**) *is assumed to be constant. This assumption is computationally efficient, which is a major consideration when evaluating tens of millions of sequences. Also, since the dispersal is purely dependent on the amino acid composition under the REM [65], and the gross features of the composition (the proportion of hydrophobic residues for example) should not change when simulating biologically realistic protein sequences, this enables the approximation *Z_fold _*≈ *S_gap_*.

The case of specific protein-protein interaction is simpler due to the lower number of states available. It was previously shown that specific binding can be adequately modeled as a combination of selection for the native ligand and against a single non-specific decoy ligand [14]. Here this approach is adopted during simulations, which are described in more detail below.

### Parameterizing the physics-based scoring functions

It has previously been shown that no single parameterization of an energy function fits all proteins optimally [34,33]. Therefore the individual weights in the scoring function were changed for each protein under investigation to maximize *Z_fold _*(maximize the specificity of sequence-structure fit in the context of alternative states). The parameter space has many dimensions, is expected to be rugged, and likely contains many local maxima. In order to sample possible parameter values efficiently, and avoid becoming trapped in the course of the search procedure, the Metropolis-Hastings Markov Chain Monte Carlo (MCMC) algorithm [66,67] was used. Acceptance probability for a move in parameter space was based on *Z_fold_*:

(16) $$p\left(\theta_{k}\right)=\left\{\begin{array}{cc} 1 & if\;Z_{fold}\left(\theta_{k}\right)>Z_{fold}\left(\theta_{k-1}\right)\\ e\frac{z_{fold}\left(\theta_{k}\right)-z_{fold}\left(\theta_{k-1}\right)}{T} & otherwise \end{array}\right)$$

where *θ_k _*is the set of weights in *V(s, c) *(eq. 1) for step *k *of the Markov chain, and *T *is the temperature of the chain. *θ_k _*is proposed by perturbing *q_k-1 _*in each dimension with values drawn from a Gaussian distribution with mean 0.1 and variance 0.1. The search space was restricted to (0,1) for all weights, and 100,000 moves were attempted at *T *= 0.1. The parameter set with the largest *Z_fold _*found during sampling was retained as the best set of weights.

To obtain the distribution of non-native scores ***G ***necessary to calculate *Z_fold _*in this procedure, a two-pronged approach was taken. The goal was to find a set of weights that makes the native sequence-structure combination as specific as possible, both with respect to the fold recognition problem (which conformation does a fixed sequence fold into?) and the inverse folding problem (which sequence best fits a fixed conformation?). Because random sequences may fold into alternative structures, the random sequences also play a role in parameterizing the fold recognition problem, where having a hydrophobic core and preferring a given backbone to an alternative state is not enough. To address specificity of conformation, 1,000 decoy conformations were generated as described above to obtain the distribution of scores ***G_struct_***. Specificity of sequence was addressed by sampling 1,000 random sequences of the same length as the native sequence (with equal probability of drawing any amino acid), threading each one through the native conformation, and obtaining from these the distribution of scores ***G_seq_***. For each score *G*, composed of individual term scores from *V(s, c) *(eq. 1), the value of each term score was re-scaled such that the overall term-specific distributions of values were of equal range. The full distribution ***G ***was formed by the union of ***G_struct _***and ***G_seq _***for each *ϴ_k_*.

When threading random sequences, or randomly perturbing the native conformation, the resulting score distribution is noticeably skewed (data not shown). In particular, the Lennard-Jones potential produces a long tail of very large scores due to steric clashes. This is not unexpected since not all structures can accommodate all sequences [68], but it does make mean and variance poor estimators of location and dispersal. Fortunately, the probability of observing a sequence *s *in a particular conformation *c *follows a Boltzmann distribution:

(17) $$p\left(\Delta G\left(s,\;c\right),\;T\right)=\frac{e^{-\Delta G\left(s,c\right)∕kT}}{\Sigma_{i}e^{-\Delta G\left(s,c_{i}\right)∕kT}}$$

where *i *runs over all possible other conformations, ΔG is the free energy of folding, *k *is Boltzmann's constant and *T *is the absolute temperature. It follows that only those alternative conformations with large negative ΔG contribute greatly to this probability, or equivalently that only those decoys with very low scores *V(s, c) *(eq. 1) are important for folding specificity. Therefore the median was used as a location estimator, and the difference between the first quartile and the median as the estimator of dispersal.

To choose the term weights for a particular protein-protein interaction, the same procedure was carried out, but only with respect to sequence specificity in one binding partner. The score distribution ***G_seq _***was generated by threading 1,000 random sequences through the constant conformation of one binding partner (while the other partner is kept constant in both sequence and structure), and scoring the resulting complex using *V(s_1_, c_1_, s_2_, c_2_) *(eq. 10) described above. In other words, *s_2 _*is randomly sampled while *s_1_*, *c_1 _*and *c_2 _*remain constant. It is then possible to calculate a gap score *Z_bind _*in the same manner as *Z_fold_*:

(18) $$Z_{bind}=\frac{\left\langle G_{bind}\right\rangle -G_{nat}^{bind}}{\sigma (G_{bind})}$$

where ***G_bind _****= ****G_seq_***. Weight parameters *w_x _*can then be chosen using the MCMC algorithm described above (eq. 16).

### Sampling sequences

Once a set of weights for *V(s, c) *(eq. 1) are obtained (the parameters *U_ab _*of *E(s, c) *in eq. 12 are fixed), the folding score gap landscape and its minima in sequence space were characterized for both scoring functions by varying *s*. Sequences were sampled based on *S_gap _*(eq. 15) both near to and far from the native state using a typical [69] MCMC approach, with acceptance probability:

(19) $$p\left(s_{k}\right)=\left\{\begin{array}{cc} 1 & if\;S_{gap}\left(s_{k}\right)>S_{gap}\left(s_{k-1}\right)\\ e\frac{s_{gap}\left(s_{k}\right)-s_{gap}\left(s_{k-1}\right)}{T} & otherwise \end{array}\right)$$

where each step *k *was a single substitution in the protein sequence.

For near-native sampling two temperatures were employed: a very high temperature for nearly unbiased sampling (*T *= 10 for the informational function *E(s, c) *of eq. 12, *T *= 50 for the physics-based function *V(s, c) *of eq. 1) and a lower temperature to find local minima (*T *= 0.1 for *E(s, c)*, *T *= 1.5 for *V(s, c)*). The low temperature was chosen to make the chain capable of passing the barrier in the energy landscape caused by an average deleterious substitution under the informational model (~0.1 units of *E(s, c)*) with a reasonably high probability (~0.1). This was also calibrated with respect to the expected fraction of sequences with better folding stability for real proteins and indirectly on dN/dS. The high temperature was chosen to make the sampling nearly independent of *S_gap _*(> 0.95 expected acceptance probability). Temperatures for the physics-based function were then chosen such that the actual acceptance frequencies obtained from sampling under the informational model were reproduced. Differences in the observed dN/dS ratios between the physics-based and informational models were due to differences in the ruggedness of the landscape and its sequence context dependence (local ruggedness).

To increase sample density, divergence of the chains from the starting state was restricted to 5%-30%, in increments of 5%, and two chains were run at each temperature and divergence limit. The number of attempted steps was adjusted to obtain ~8,500 unique samples for each function. Samples were projected onto a two-dimensional representation of sequence space using Sammon mapping [70] and score surfaces were calculated using Gnuplot v 4.2.

To discover regions of the landscape where most mutations are destabilizing (i.e *S_gap _*is near a maximum), an important feature of biological protein sequences leading to observed dN/dS ratios, ten replicate chains attempting 100,000 steps were run for each function at the lower sampling temperature without any divergence restrictions. The chains were thinned by taking every fourth unique sample and the final 100 such samples were retained, resulting in 1,000 unique samples for each scoring function. Samples were Sammon-mapped and sequence logos [71] were calculated to assess convergence. Structural properties of the sampled equilibria were further characterized by projecting the consensus sequence at each position with > 2 bits of information onto the protein conformation.

### Measuring scoring performance

To test the accuracy of the scoring functions, *Z_fold _*was calculated for the native sequence and 1,000 random sequences. The test was performed on the same diverse set of structures used in the development of the side chain replacement method employed [35].

The interaction score was tested by a similar scoring procedure. In this case a subset of the structures from the PepX database [72], which describes protein-peptide interactions, was constructed. As for the folding score test set, 100 structures (25 from each major SCOP Class) were selected, each a centroid of a cluster of binding interfaces at the 3 Å-75% level of PepX. For each Class with more than 25 available centroids, the structures were sorted by average B-factor across the binding interface, and the 25 structures with the lowest B-factor were selected. A list of these structures can be found in Additional File 1. *Z_bind _*was calculated as described above (eq 18) for the native peptide sequence and a set of 1,000 random peptide sequences for each complex.

In addition, the distribution of weights for each term in *V(s, c) *(eq. 1) and *V(s_1_, c_1_, s_2_, c_2_) *(eq. 10) across the structural data sets was generated to compare the relative importance of the terms. The effect on the gap score of using each term individually was also examined. Furthermore, the numerical stability of the MCMC algorithm for choosing weights and the characteristics of the parameter-stability landscape were probed by producing eight different sets of non-native scores ***G ***for the SAP protein, and re-running the parameterization 100 times for each set.

### Simulating sequences

As a test of the utility of the methods in an evolutionary context, simulations were performed under negative selection for protein folding and binding. In a manner similar to that of Rastogi et al. [11], a population of 1,000 virtual organisms containing a single copy of the SAP protein [25] (PDB ID: 1D4T) was evolved for 200,000 generations at a rate of 10^-5 ^mutations per bp per generation at the DNA level, with transitions being twice as probable as tranversions. To obtain a thermodynamically stable starting point, non-native optima of *S_gap _*(eq. 15) in protein sequence space were sampled as described above and a starting DNA sequence was randomly selected from the reverse translation of those samples.

In each generation, the mutated DNA sequence in each organism was translated to protein, threaded through the native SAP conformation *c_protein_*, and the fitness calculated as

(20) $$w\left(s\right)=\left\{\begin{array}{cc} 0 & if\;S_{gap}\left(s\right)<S_{gap}^{start}or\;V_{bind}\left(s\right)>V_{bind}^{start}\\ 0.9 & if\;S_{gap}\left(s\right)\geq S_{gap}^{start}and\;V_{bind}\left(s\right)\leq V_{bind}^{start}and\;V_{decoy}\left(s\right)\leq V_{bind}^{start}\\ 1 & otherwise \end{array}\right)$$

where *S_gap_(s) *is the fold gap score (eq. 15) of the current sequence *s*, *S_gap_^start ^*is the fold gap score of the starting sequence, *V_bind_(s) *is the binding score *V(s, c_protein_, s_ligand_, c_ligand_) *(eq. 10), *s_ligand _*and *c_ligand _*correspond to the SLAM peptide (the native ligand of SAP), *V_bind_^start ^*is the binding score of the starting protein sequence to the same ligand, and *V_decoy_(s) *is the binding score *V(s, c_protein_*, *s_decoy_, c_ligand_) *for a decoy ligand. Simulations under the informational model used *E(s, c) *(eq. 12) to compute *S_gap _*and *E(s_1_, c_1_, s_2_, c_2_) *(eq. 13) to compute binding scores. In other words, mutations that make the protein or the protein-ligand complex less stable than the starting point are infinitely deleterious, mutations that maintain those stabilities but enable binding of the decoy ligand are somewhat deleterious, and all other changes are neutral. Organisms were then propagated to the next generation by random sampling weighted by fitness *w *(eq. 20), with replacement, while maintaining a constant population size.

The decoy ligand were constructed by threading a sequence *s_decoy _*= IWMTIYMIIIT through the SLAM peptide conformation *c_ligand _*for the informational function, and *s_decoy _*= RLPTIYICITG for the physics-based function. Both sequences are variations on the experimentally determined XXXTIYXX(VI)XX SAP-binding motif [25], and do not bind the protein at the starting point of the simulation.

Finally, each simulation was replicated 10×, and the resulting sequences analyzed. Structural patterns of evolutionary rates (dN/dS) were measured by comparing sequences to the original sequence and calculating according to the PBL method [73,16], and the distribution of position-specific residue preference was compared with that in the Pfam database [30] for the same fold by constructing sequence logos [71] from the evolved sequences. The sequences were also tested for emergence of rate heterogeneity by assessing the superior fit of the data to the rates-across-sites model over an equal-rates model using ProtTest [74]. To discern if the novel solvation model preserves the general pattern of hydrophobic core and hydrophilic surface the proportion of hydrophobic and hydrophilic residues in parts of the protein were measured before and after simulation.

### Availability

The scoring function and simulation software are available as C++ code by request. The code will be released as part of open source software for sequence simulation to be described in a future publication.

## Authors' contributions

JAG contributed to the design of the analysis, wrote simulation software, performed all analysis presented using the software, and co-wrote the manuscript. PN contributed to writing simulation software used in this study. JK contributed to the design of the analysis. DAL conceived of the study, contributed to the design of the analysis, and co-wrote the manuscript. All authors read and approved the final manuscript.

## Supplementary Material

Additional file 1 **Table S1**. The list of complexes used for testing the binding scoring functions is shown. The PDB accession number, the SCOP Class the complex belongs to, the corresponding Uniprot sequence (if any), and the long protein name from Uniprot (if any) are listed. Click here for file
